# Testing the combination of Feeling Safe and peer counselling against formulation-based cognitive behaviour therapy to promote psychological wellbeing in people with persecutory delusions: study protocol for a randomized controlled trial (the Feeling Safe-NL Trial)

**DOI:** 10.1186/s13063-023-07661-x

**Published:** 2023-10-05

**Authors:** Eva Tolmeijer, Felicity Waite, Louise Isham, Laura Bringmann, Robin Timmers, Arjan van den Berg, Hanneke Schuurmans, Anton B. P. Staring, Paul de Bont, Rob van Grunsven, Gert Stulp, Ben Wijnen, Mark van der Gaag, Daniel Freeman, David van den Berg

**Affiliations:** 1 Department of Clinical Psychology, VU University and Amsterdam Public Health Research, Amsterdam, The Netherlands; 2 Department of Psychosis, Parnassia Psychiatric Institute, The Hague, The Netherlands; 3 Department of Experimental Psychology, University of Oxford and Oxford Health NHS Foundation Trust, Oxford, UK; 4 Department of Psychometrics and Statistics, University of Groningen, Groningen, The Netherlands; 5 Voice-Hearing Support and Recovery-Team, RIBW Nijmegen and Rivierenland, Nijmegen, The Netherlands; 6 University of Applied Sciences Nijmegen, Nijmegen, The Netherlands; 7 Department of Health, Wellbeing and Sport, Zadkine College Rotterdam, Rotterdam, The Netherlands; 8 Mental Health Organizations Oost Brabant, Boekel, The Netherlands; 9 ABC Department for First Episode Psychosis, Altrecht Psychiatric Institute, Utrecht, The Netherlands; 10 Department of Sociology, University of Groningen, Groningen, The Netherlands; 11 Centre of Economic Evaluation and Machine Learning, Trimbos Institute (Netherlands Institute of Mental Health and Addiction), Utrecht, The Netherlands

**Keywords:** Paranoia, Persecutory delusions, Psychosis, Peer counselling, Cognitive behaviour therapy, Randomized controlled trial

## Abstract

**Background:**

Persecutory delusions are strong threat beliefs about others’ negative intentions. They can have a major impact on patients’ day-to-day life. The Feeling Safe Programme is a new translational cognitive-behaviour therapy that helps patients modify threat beliefs and relearn safety by targeting key psychological causal factors. A different intervention approach, with growing international interest, is peer counselling to facilitate personal recovery. Combining these two approaches is a potential avenue to maximize patient outcomes. This combination of two different treatments will be tested as the Feeling Safe-NL Programme, which aims to promote psychological wellbeing. We will test whether Feeling Safe-NL is more effective and more cost-effective in improving mental wellbeing and reducing persecutory delusions than the current guideline intervention of formulation-based CBT for psychosis (CBTp).

**Methods:**

A single-blind parallel-group randomized controlled trial for 190 out-patients who experience persecutory delusions and low mental wellbeing. Patients will be randomized (1:1) to Feeling Safe-NL (Feeling Safe and peer counselling) or to formulation-based CBTp, both provided over a period of 6 months. Participants in both conditions are offered the possibility to self-monitor their recovery process. Blinded assessments will be conducted at 0, 6 (post-treatment), 12, and 18 months. The primary outcome is mental wellbeing. The overall effect over time (baseline to 18-month follow-up) and the effects at each timepoint will be determined. Secondary outcomes include the severity of the persecutory delusion, general paranoid ideation, patient-chosen therapy outcomes, and activity. Service use data and quality of life data will be collected for the health-economic evaluation.

**Discussion:**

The Feeling Safe-NL Trial is the first to evaluate a treatment for people with persecutory delusions, while using mental wellbeing as the primary outcome. It will also provide the first evaluation of the combination of a peer counselling intervention and a CBT-based program for recovery from persecutory delusions.

**Trial registration:**

Current Controlled Trials ISRCTN25766661 (retrospectively registered 7 July 2022).

## Administrative information

Note: the numbers in curly brackets in this protocol refer to SPIRIT checklist item numbers. The order of the items has been modified to group similar items (see http://www.equator-network.org/reporting-guidelines/spirit-2013-statement-defining-standard-protocol-items-for-clinical-trials/).

**Table:** 

Title {1}	Testing the combination of Feeling Safe and peer counselling against formulation-based cognitive behaviour therapy to promote psychological wellbeing in people with persecutory delusions: study protocol for a randomized controlled trial (the Feeling Safe-NL Trial).
Trial registration {2a and 2b}.	ISRCTN registry (https://doi.org/10.1186/ISRCTN25766661)
Protocol version {3}	Protocol version 2 (21–12-2022).
Funding {4}	Funded by the independent and self-governing organization ZonMw for healthcare research.
Author details {5a}	Eva Tolmeijer^1,2^ Felicity Waite^3^ Louise Isham^3^ Laura Bringmann^4^ Robin Timmers^5,6^ Arjan van den Berg^7^ Hanneke Schuurmans^8^ Anton B.P. Staring^9^ Paul de Bont^8^ Rob van Grunsven^2^ Gert Stulp^10^ Ben Wijnen^11^ Mark van der Gaag^1^ Daniel Freeman^3^ David van den Berg^1,2^ ^1^Department of Clinical Psychology, VU University and Amsterdam Public Health Research. ^2^Department of Psychosis, Parnassia Psychiatric Institute, The Hague. ^3^Department of Experimental Psychology, University of Oxford and Oxford Health NHS Foundation Trust. ^4^Department of Psychometrics and Statistics, University of Groningen. ^5^Voice-hearing support and recovery-team, RIBW Nijmegen and Rivierenland. ^6^University of Applied Sciences Nijmegen. ^7^Department of Health, Wellbeing and Sport, Zadkine College Rotterdam. ^8^Mental health organizations Oost Brabant. ^9^ABC Department for First Episode Psychosis, Altrecht Psychiatric Institute. ^10^Department of Sociology, University of Groningen. ^11^Centre of Economic Evaluation and Machine Learning, Trimbos Institute (Netherlands Institute of Mental Health and Addiction).
Name and contact information for the trial sponsor {5b}	VU University Amsterdam, Faculty of Behavioral and Movement Sciences, Department of Clinical Psychology Van der Boechorststraat 7 1081 BT Amsterdam.
Role of sponsor {5c}	The sponsor and funding body have no role in the design of the study and collection, analysis, and interpretation of data and in writing the manuscript.

## Introduction

### Background and rationale {6a}

Persecutory delusions are strong threat beliefs with specific content about others’ negative intentions. They are common and transdiagnostic experiences in people with severe mental health problems [1], and they are associated with impairments in multiple functional domains. About half of patients with persistent persecutory delusions experience psychological wellbeing scores in the lowest 2% of the general population [2]. Considering the impact of persecutory delusions on patients' daily life, it is crucial that they receive treatment. Effective treatments include medication and formulation-based cognitive behaviour therapy for psychosis (CBTp) [3, 4]. Yet the effect sizes for those two treatments are small to moderate and treatment outcomes can be improved [3].

The new translational Feeling Safe Programme, a targeted psychological treatment addressing key contributory causal factors, has demonstrated large treatment effects for persecutory delusions (*d* = 1.2 when compared to an active psychological control condition), the largest treatment effects for persecutory delusions to date [5]. Besides advances in psychological therapy, there is an internationally growing trend to adopt peer counselling in mental healthcare [6, 7] with evidence for positive effects on personal recovery [8, 9]. Both clinical and personal recovery independently and synergistically contribute to improved mental wellbeing [10]. Uniquely bringing together these two promising approaches, Feeling Safe and peer counselling — two different types of interventions, delivered by different staff members at the same time and in collaboration, has the potential to maximize patients’ recovery and mental wellbeing. This combination will be called ‘The Feeling Safe-NL Programme’ and our aim is to provide an evaluation against the psychological guideline intervention of formulation-based CBTp.

#### The Feeling Safe Programme

Research has shown that there are several common empirical maintenance factors of inaccurate threat beliefs that patients frequently wish to be treated [11]. The Feeling Safe Programme is the culmination of over a decade of research and involves a translational approach in which five psychological maintenance factors of threat beliefs (worry, low self-esteem, anomalous experiences, insomnia, and safety-seeking behaviours or defences) are identified and addressed with CBT modules of approximately six to eight sessions [5]. These brief CBT modules have been specifically adapted for patients with persecutory delusions and have been tested in separate randomized controlled trials. Therapy modules include: improving sleep [12], increasing self-confidence [13], reducing worry [14], reducing the impact of voices, and feeling safe enough [15]. The Feeling Safe Programme is an individual therapy of approximately 20 sessions in which on average three therapy modules are completed, including the core module focused on rediscovering safety by reducing safety-seeking behaviours or defences and building experiences of being safe enough [5]. For each person it is first determined which of the maintenance factors are contributing to sustaining their fear of others. This results in a personalized treatment menu. The therapist consults, but the patient decides what modules to do and in what order, giving the patient control from the outset. As a result, brief CBT modules are delivered that match the persons’ experiences and personal choice: e.g. ‘I want to sleep better’ or ‘I want to worry less’. The strategy is to address maintenance factors one-by-one, then enter previously avoided situations to enable the person to learn they are safe enough. Through helping people to re-engage with activities while gradually lowering their defences people build new memories of being safe enough. The overarching goals are to feel safer, happier, and get people back to doing what they want to do. These goals and the overall program were positively evaluated by patients [16] and resulted in large effect size improvements in persecutory beliefs and wider benefits including improvements in mental wellbeing and participation in day-to-day activities [5].

#### Peer counselling

Recovery from severe mental health problems is a complex and personal process and peer counselling is referred to as the help and support of people with lived experience of mental health problems to navigate this process [6]. The foundation of peer counselling is that people who have faced, endured and overcome adversity can offer hope, emotional support and experiential knowledge (i.e. knowledge gained through experience) to facilitate recovery [17]. Peer-counselling is one of the roles of experts by experience, which refers to people who are able to share learning in different contexts based on their experiential knowledge. Peer counsellors can promote people’s recovery in mental healthcare settings through reciprocal sharing of experiences, identifying meaning in past and current experiences, and by promoting experiential knowledge through helping people to reflect on their experiences and strengths. Peer counsellors are increasingly involved in delivering mental healthcare [6, 7], and in The Netherlands, peer counsellors have a professional register, code and competence profile [18, 19]. A recent review on the effectiveness of peer counselling reported small positive effects on clinical recovery, overall personal recovery and more specifically hope, in particular for people with severe mental illness [8]. Qualitative research also reveals the instalment of hope as a recurring theme, alongside other themes including reciprocal sharing of experiences, both contributing to the central concept of de-stigmatization [20]. For one-to-one peer support, specifically, psychosocial benefits have been found including improvements in self-reported recovery and empowerment [9]. Also in an experimental setting, research has shown a benefit of delivering recovery-oriented messages by peers [21]. Postulated mechanisms of peer support include sharing of lived experience, emotional engagement, the liminal position between mental healthcare staff and patients, strengths-focus and reducing internal stigma [22]. Some of these unique aspects of peer counselling are promising for promoting recovery and mental wellbeing in patients with persecutory delusions.

#### The Feeling Safe-NL Programme

The combination of Feeling Safe and peer counselling moves towards a dual approach to therapy. Therapists work on reducing the maintenance factors of inaccurate threat beliefs (persecutory delusions) using brief CBT modules and supporting patients to relearn safety. At the same time, professional peer counsellors focus on addressing personal recovery by sharing of experiences, identifying meaning in past and current experiences, and helping people to build experiential knowledge. Besides the close collaboration between therapists and peer counsellors, Feeling Safe-NL offers a trauma-imagery module based on the evidence for trauma imagery as a treatable psychological causal factor of threat beliefs [23, 24]. The trauma imagery module replaces the reasoning biases module, as this module was rarely chosen and the reasoning biases factor is also implicitly addressed in other therapy modules [5]. Additionally, the program offers people the possibility to self-monitor change during their recovery process since this has been found to promote shared decision-making, agency [25] and motivation [26]. Together, the Feeling Safe-NL Programme provides a clear framework for tackling complex problems: install hope and build experiential knowledge while reducing the maintenance factors of persecutory delusions one-by-one, then with support re-enter situations, directly re-learn safety and work towards personal recovery goals.

#### The Feeling Safe-NL Trial

This randomized controlled trial will recruit outpatients with persecutory delusions, independent of the Diagnostic and Statistical Manual of Mental Disorders (DSM)-classification. The primary hypothesis is that the Feeling Safe-NL Programme will lead to greater improvement in mental wellbeing over time (from baseline to 18-month follow-up) than formulation-based CBTp. The secondary hypotheses are that the Feeling Safe-NL Programme will be more effective than formulation-based CBTp over time (from baseline to 18-month follow-up) in 1) reducing the severity of the persecutory delusion, 2) reducing general paranoid ideation, 3) improving patient-chosen outcomes of therapy and 4) improving activity levels. We also hypothesize that the Feeling Safe-NL Programme will be more cost-effective than formulation-based CBTp (from baseline to 18-month follow-up). The trial will also include an explanatory component. We hypothesize that changes in the key maintenance factors (e.g. worry, low self-esteem, anomalous experiences, insomnia, safety-seeking behaviours or defences, and trauma imagery), resilience, and personal recovery will mediate changes in mental wellbeing and persecutory delusions. We will also record all service use, and other relevant health economic data, for the health-economic evaluation.

### Objectives {7}

The primary objective is to test whether the Feeling Safe-NL Programme is more effective in improving mental wellbeing over time than formulation-based CBTp. The overall effect over time (baseline to 18-month follow-up) and the effects at each timepoint will be determined [27].

The secondary objectives are to test whether the Feeling Safe-NL Programme is more effective than formulation-based CBTp over time in (1) reducing the severity of the persecutory delusion, (2) reducing general paranoid ideation, (3) improving patient chosen outcomes of therapy, and (4) improving activity levels. Additionally, we investigate the mediators of improved mental wellbeing and reduced persecutory delusions and whether the Feeling Safe-NL Programme is more cost-effective than formulation-based CBTp.

### Trial design {8}

**Fig. 1:**
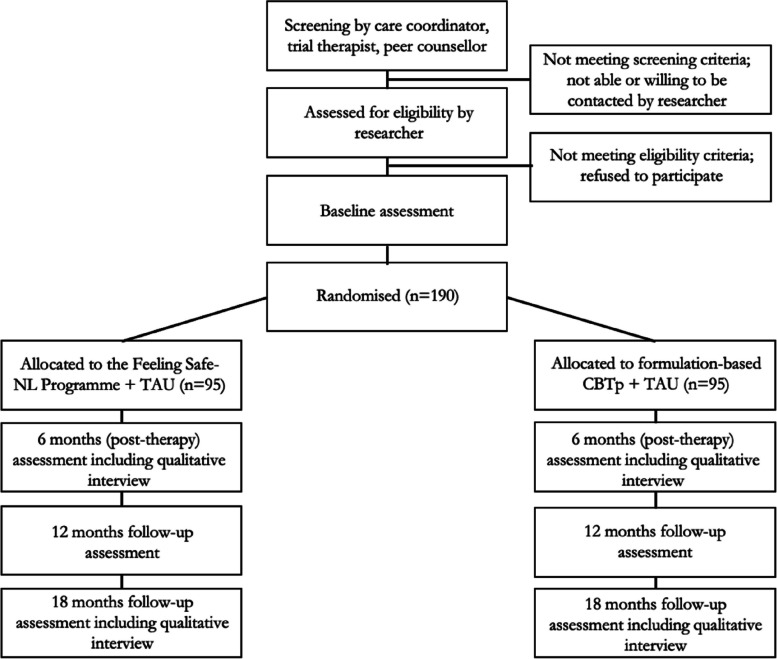
Trial flow diagram

A pragmatic single-blind superiority randomized controlled trial with two parallel groups. Patients will be randomized (1:1) to the Feeling Safe-NL Programme or formulation-based CBTp. Measurements are conducted at baseline, 6-, 12-, and 18-month follow-up (see Fig. 1). In both arms, people will receive weekly therapy sessions over a period of 6 months. To support the therapies in both arms, optional self-monitoring is available via brief questionnaires of which the outcomes are visualized. This supports the participant and therapist in monitoring change and shared decision-making.

## Methods: participants, interventions and outcomes

### Study setting {9}

Participants will be recruited from outpatient services of several large mental health organizations in the Netherlands (Parnassia Groep, Altrecht and GGZ Oost Brabant) that offer care to people with persecutory delusions.

### Eligibility criteria {10}

We will recruit outpatients with persecutory delusions, independent of DSM classification. The inclusion criteria are main persecutory belief held with at least 60% conviction; low mental wellbeing (score ≤ 43 on the Warwick Edinburgh Mental Well-Being Scale [28], determined using clinical cut-off calculation using the Oxford Feeling Safe sample [5]); and age ≥ 16 years. The exclusion criteria are insufficient competence in the Dutch language to participate in research; currently receiving individual therapy or peer counselling with a frequency of at least one session per month; and unable to understand and sign the informed consent form.

### Who will take informed consent? {26a}

A two-step consent procedure will be used that allows identifying those people that could benefit from the target interventions. First, a local trial therapist or care coordinator asks patients with persecutory delusions for written permission to provide their contact details to the trial assessors so they can provide more information about the study. Second, the patient will be contacted by a trial assessor and informed about the study, both verbally and in writing. Patients then receive at least one week to consider participation in the study. If patients decide to participate, they are invited for a face-to-face meeting in which they can ask additional questions about participation, informed consent is obtained, and inclusion and exclusion criteria are assessed. Eligible participants will be seen for the baseline assessment after which they will be randomized to either Feeling Safe-NL or formulation-based CBTp.

### Additional consent provisions for collection and use of participant data and biological specimens {26b}

N/a. No data are collected for which additional consent provisions are required.

## Interventions

### Explanation for the choice of comparators {6b}

We will compare the Feeling Safe-NL Programme to the international guideline psychological intervention formulation-based CBTp [29, 30]. This will allow us to investigate if the Feeling Safe-NL Programme, involving modular CBT and peer counselling, is more effective than the guideline psychological intervention in the long-term.

### Intervention description {11a}

#### The Feeling Safe-NL Programme

Feeling Safe-NL builds on the Feeling Safe Programme [5] with three adaptions. First, Feeling Safe-NL involves close collaboration between therapists and professional peer counsellors. While therapists work on reducing the maintenance factors of persecutory delusions using targeted CBT and supporting patients to relearn safety, professional peer counsellors focus on addressing personal recovery, together promoting mental wellbeing. Second, Feeling Safe-NL includes trauma imagery as a treatable maintenance factor of threat beliefs [23, 24]. Third, Feeling Safe-NL offers the possibility to self-monitor change to promote people’s agency, shared decision-making and motivation [25, 26]. Feeling Safe-NL involves approximately 20 weekly therapy sessions and 10 bi-weekly individual peer counselling meetings. There are also collaborative meetings (patient, peer counsellor, therapist) to evaluate progress and enhance synergy. This is done at least at the start of the program, near the end of each CBT module and at the end of the program.

**Fig. 2:**
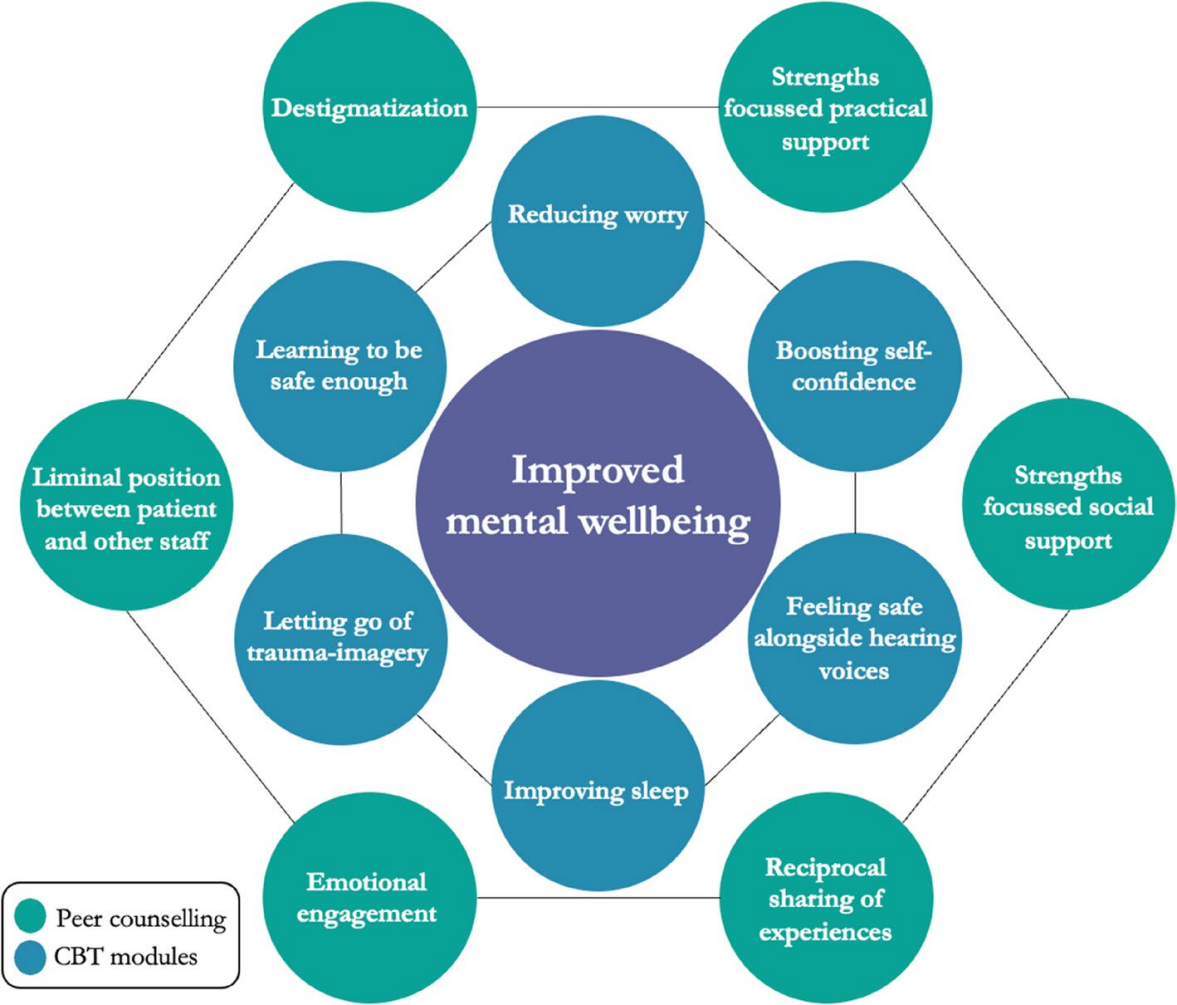
The dual approach to improving mental wellbeing in the Feeling Safe-NL Programme: relearn safety by offering targeted CBT modules while promoting personal recovery through postulated mechanisms of peer counselling [5, 22–24]

Following an introductory meeting with both the therapist and peer counsellor, the therapist and peer counsellor assess the maintenance factors and wishes and needs of the person, respectively. Following this assessment, the therapist offers the patient a menu of relevant CBT modules. Typically, three to four modules are completed, based on patient choice. Modules that can be offered are reducing worry, boosting self-confidence, feeling safe alongside hearing voices, improving sleep, letting go of trauma imagery, and learning to be safe enough through behavioural tests for building safety beliefs. Concurrent to the therapy, the peer counsellor and participant work together according to the sponsor model [31]; the peer counsellor supports the participant during the Feeling Safe-NL Programme based on the participants’ wishes and needs, considering possibilities. The participant and peer counselor work together on personal recovery including identifying and using strengths [22]. The dual approach to improving mental wellbeing is presented in Fig. 2.

#### Formulation-based CBTp

CBTp will follow the guideline formulation-based manual for persecutory delusions in the Netherlands [32], which is an updated version of the British manuals, e.g [33]. including some options for modular CBT interventions (e.g. competitive memory training for increasing self-confidence, CBT for insomnia, and meta-cognitive training for worry) [34–39] and trauma-focused CBT interventions [40] depending on the psychological case-formulation. Influenced by the earlier development studies for Feeling Safe, there is therefore a degree of overlap in therapy content. The formulation-based CBTp manual for persecutory delusions consists of four phases: introduction, formulation and goal setting, intervention and change techniques, and consolidation. The aim is to reduce the negative behavioural and emotional impact of persecutory delusions, to promote more helpful information processing, perspectives, and behavioural coping strategies and to work towards personal therapy goals.

Both therapies will be delivered by therapists with at least an MSc in Clinical Psychology and 100 hours of accredited CBT training. All therapists will be trained in and deliver both interventions (to eliminate therapist effects). Therapists will receive weekly supervision from a clinical psychologist and CBT supervisor. Therapists start seeing participants for the trial after four recorded therapy sessions (two for Feeling Safe-NL and two for formulation-based CBTp) that have been rated and scored ≥ 4 on each relevant item of the Cognitive Therapy Rating Scale-Revised for Psychosis. Professional peer counsellors meet the standards of the Dutch professional competence profile [18, 19] and receive additional training in strengths-based methodologies [41] and promoting experiential knowledge regarding the six empirical maintenance factors being addressed by the Feeling Safe therapy modules. Professional peer counsellors start seeing participants after their written and practical assignments reflect the list of competencies in the professional competence profile.

### Criteria for discontinuing or modifying allocated interventions {11b}

Interventions will be discontinued when a participant no longer wants to continue the intervention or when a participant is an early therapy completer. The interventions that are tested are found to be feasible and safe in this population and the study is situated in routine clinical practice.

### Strategies to improve adherence to interventions {11c}

Both formulation-based CBTp and Feeling Safe-NL involve shared decision-making and tailoring of CBT-techniques to each individual patient to promote adherence and outcomes. Therapists and peer counsellors generally see participants at their local community mental health team, but home visits are possible to improve adherence to the interventions.

### Relevant concomitant care permitted or prohibited during the trial {11d}

Standard care will continue as usual and will be monitored by the research team.

### Provisions for post-trial care {30}

There are no provisions for post-trial care since the interventions are found to be feasible and safe in this population and the study is situated in routine clinical practice. Usual care will continue when the trial ends.

### Outcomes {12}

The primary outcome will be psychological wellbeing (Warwick-Edinburgh Mental Well-Being Scale, WEMWBS)^28^ over time (assessed using the overall effect over time from baseline to 18-month follow-up and the effects at each timepoint) [27].

The secondary outcomes include persecutory delusion severity (Psychotic Symptom Rating Scale-Delusion Subscale, PSYRATS-DS) [42], general paranoid ideation (Revised-Green et al. Paranoid Thoughts Scale, R-GPTS), patient chosen outcomes (CHoice in Outcome In Cognitive behaviour therapy for psychosEs, CHOICE) and activity (time budget). Outcomes for conviction of the main persecutory delusion will also be dichotomized in line with the Feeling Safe Study (in which recovery is defined as conviction falling below 50%) [5].

We will also assess several mediators: trauma imagery (Trauma Screening Questionnaire, TSQ; PTSD Checklist for DSM-5, PCL-5) [43–45], insomnia (Insomnia Severity Index, ISI) [46], self-esteem (Brief Core Schema Scales, BCSS) [47], worry (Dunn Worry Questionnaire) [48], anomalous experiences (Voices Impact Scale, VIS; a publication concerning the psychometrics is underway), safety-seeking behaviours or defences (Oxford Agoraphobic Avoidance Scale, O-AS [49]; Oxford Paranoia Defence Behaviours Questionnaire, OPDBQ; Lambe et al., *in prep*), reasoning biases (Fast and Slow Thinking Questionnaire, FaST; Explanations of Experiences, EofE, Maudsley Assessment of Delusions Schedule-Possibility of being Mistaken, MADS-PM) [50–52], personal recovery (Questionnaire about the Process of Recovery, QPR) [53], and resilience (The Brief Resilience Scale, BRS) [54].

Other study parameters include the therapeutic relationship (Counsellor Rating Form-Short, CRF-S) [55] and working alliance (Working Alliance Inventory-Short Form Revised, WAI-SR) [56]. For the health-economic evaluation we will use the Treatment Inventory of Costs in Patients with psychiatric disorders (TIC-P) and the standardized EuroQol  5-Dimension 5-Level questionnaire (EQ-5D-5L) at baseline and after 6 (post-intervention) 12 and 18 months.

### Participant timeline {13}

Following enrolment, participants will be allocated to formulation-based CBTp or the Feeling Safe-NL Programme. Assessments will be carried out at baseline and after 6 (post-intervention) 12 and 18 months. A schematic diagram is provided in Table 1.

**Table 1:**
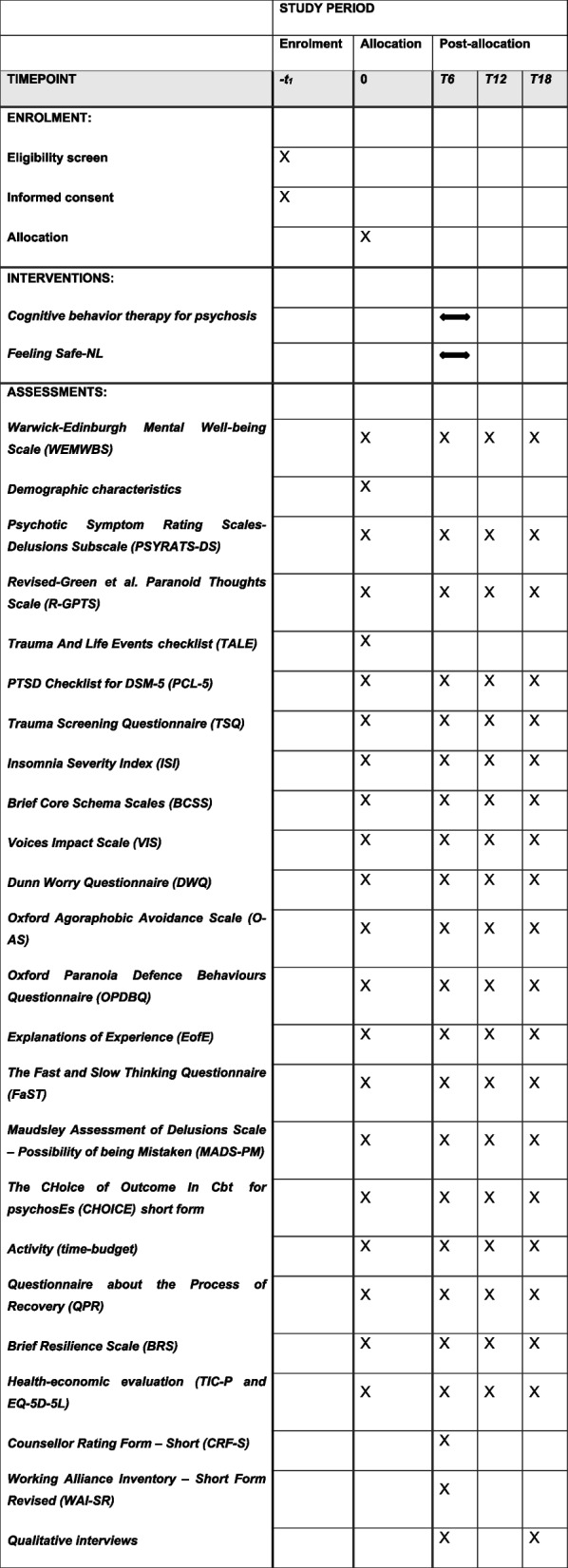
Overview of the procedures and instruments used at the different time points

### Sample size {14}

The sample size for longitudinal intention-to-treat (ITT) analyses with linear mixed models (LMM) was calculated according to the method presented by Liu and Liang (1997) and based on data from previous RCTs [5, 13–15, 24]. Although an ITT analysis with LMM is robust against moderate attrition, we aim to include an additional 10% to compensate for potential dropout. With alpha = 0.05; beta = 0.2; rho = 0.5; 3 repeated follow-up assessments and 10% drop-out; we will need 95 participants in each arm to detect small to medium effects (0.35). Therefore, we aim to include 190 participants.

### Recruitment {15}

Our strategy for achieving adequate participant enrolment to reach the target sample size is to recruit at several large mental healthcare institutions that collaboratively offer care to more than 200,000 patients. Additionally, we work with a committed group of peer counsellors and therapists and trained trial assessors who are involved in informing patients and community health team staff.

## Assignment of interventions: allocation

### Sequence generation {16a}

Patients will be randomized by our independent randomization bureau using a scientific randomization program on the internet (http://www.randomizer.org). The allocation sequence ensures an equal assignment to both interventions for each site.

### Concealment mechanism {16b}

The independent randomization bureau is the only staff with access to the randomization sequence, and they will keep this concealed from all others. Therefore, researchers and therapists cannot know beforehand to which group a new included participant will be allocated.

### Implementation {16c}

Trial assessors will enrol participants in the trial. Our independent randomization bureau will generate the allocation sequence and assign participants to the interventions. After randomization of a new participant, trial therapists receive an email from the randomization bureau with the allocated intervention. The therapists inform the participant about the allocation and start the therapy.

## Assignment of interventions: blinding

### Who will be blinded {17a}

The trial assessors will be blind to group allocation, but participants, peer counsellors, and therapists will not be.

### Procedure for unblinding if needed {17b}

In case of unblinding during an assessment by a participant, the assessment is stopped and finished by another trial assessor. The unblinded trial assessor will also not execute future assessments with the specific participant. The number of unblinding incidents will be registered.

## Data collection and management

### Plans for assessment and collection of outcomes {18a}

Assessments will be conducted by trained trial assessors to promote data quality. Table 1 presents an overview of the instruments used.

#### Demographic characteristics: to determine demographics at recruitment

These include age, gender, cultural identity, education, employment, living condition, relationship status, duration of being in mental healthcare and previous experience with CBT.

#### WEMWBS: to assess mental wellbeing

The Warwick-Edinburgh Mental Well-Being Scale (WEMWBS)^28^ is a 14-item self-report questionnaire covering both feeling and functioning aspects of mental wellbeing. The scale has been widely used for monitoring and investigating the determinants of mental wellbeing. The WEMWBS has excellent psychometric properties in terms of reliability and validity.

#### PSYRATS: to assess persecutory delusions

The Psychotic Symptom Rating Scales-Delusions Subscale (PSYRATS-DS)^42^ consists of six items which assess the following dimensions of delusions: frequency of preoccupation with delusions, duration of preoccupation with delusions, conviction, frequency of distress, intensity of distress and disruption to life caused by beliefs. Test–retest reliability has been found to be high [57]. Scores on the PSYRATS can be used to calculate factors relating to frequency and distress, which have a reported ICC of 0.93 and 0.87, respectively [58].

#### R-GPTS: to assess paranoid ideation

The Revised-Green et al., Paranoid Thoughts Scales (R-GPTS) is an 18-item self-report questionnaire that assesses ideas of persecution (10-item) and social reference (8-item) and was found to have excellent psychometric properties [59]. A hierarchical relationship between social reference and persecution was found.

#### Time-budget measure: to measure activity

The time budget measure is used to assess (social) activity. The measure is specifically designed for people experiencing psychosis [60].

#### CHOICE: to assess patient satisfaction

The CHoice of Outcome In Cbt for psychosEs (CHOICE) short form [61] is a 12-item self-report questionnaire examining satisfaction with therapy using patient definitions of recovery and therapy aims.

#### TIC-P and EQ-5D-5L: to assess resource use and quality-adjusted life years

Resource use will be measured with the Trimbos Institute and iMTA Cost Questionnaire for Psychiatry (TIC-P), short version. Using the TIC-P, we will measure healthcare usage, patients’ and their family’s out-of-pocket costs, and productivity losses owing to absenteeism and presenteeism. The EuroQol 5-dimension 5-level questionnaire (EQ-5D-5L) will be used to measure health-related quality of life (HRQoL). The EQ-5D-5L describes health states over five domains of mobility, self-care, usual activities, pain/discomfort, and anxiety/ depression. Dutch tariffs [62] will be applied to each of the health states to obtain utilities, in which 0 represents death and 1 full health. Next, utilities will be combined over time by calculating the area under the curve (or linear interpolation) to calculate quality-adjusted life years (QALYs).

#### TALE: to assess past-traumatic experiences at recruitment

The Trauma And Life Events checklist (TALE) is a 21-item checklist for traumatic experiences and shocking life events in people with psychotic disorders that was recently developed [63]. It covers all main categories of trauma (abuse and neglect) and bullying, stressful psychotic experiences, experiences of loss, and stressful experiences in contact with mental health or criminal justice services.

#### CRF-S: to assess the participant’s view of the therapist

The Counsellor Rating Form – Short (CRF-S) [55] is a 12-item self-report questionnaire that assesses the counsellor’s attractiveness, expertness and trustworthiness as perceived by the participant on a 7-point Likert scale ranging from 1 (‘not at all’) to 7 (‘very’). The CRF-S has been shown to be psychometrically robust.

#### WAI-SR: to assess therapeutic alliance

The Working Alliance Inventory – Short Form Revised [56] (WAI-SR) is a 12-item self-report questionnaire that assesses the strength of the therapeutic alliance on a 5-point Likert ranging from 1 (‘rarely or never’) to 5 (‘always’). The WAI-SR has been shown to have high construct validity, test–retest reliability, and internal consistency [56].

#### TSQ: to screen for PTSD diagnoses

The Trauma Screening Questionnaire (TSQ) is a 10-item self-report questionnaire that assesses post-traumatic stress symptoms [43]. The scale has five re-experiencing items and five arousal items. A score ≥ 6 on the TSQ is indicative of the probability of PTSD diagnosis in people with psychosis [44].

#### PCL-5: to assess the severity of PTSD symptoms

The PTSD Checklist for the DSM-5 (PCL-5) is a 20-item self-report questionnaire assessing PTSD symptoms. The questionnaire has been validated in many different types of samples [64]. The DSM-5 version was recently found to demonstrate strong validity and reliability [45]. PCL-5 item scores are summed to yield a continuous PTSD symptom severity score.

#### ISI: to assess insomnia

The Insomnia Severity Index (ISI) is a 7-item self-report questionnaire that assesses the severity of both night-time and daytime components of insomnia [46].

#### BCSS: to assess beliefs about self and other people

The Brief Core Schema Scales (BCSS) is a 24-item self-report questionnaire that assesses schemata concerning self and other people [47]. The questionnaire assesses four dimensions of self and other evaluation: negative-self, positive-self, negative-other, positive-other.

#### DWQ: to assess worry

The Dunn Worry Questionnaire (DWQ) is a 10-item questionnaire that assesses general problematic worry [48]. The scale has demonstrated excellent internal reliability, test–retest reliability, concurrent validity, and sensitivity to change.

#### VIS: to assess the impact of auditory verbal hallucinations

The Voice Impact Scale (VIS) is a 24-item self-report questionnaire that assesses the impact of voices. It contains three 8-item subscales: negative impact of voices, positive impact of voices, and living well with voices. The psychometric qualities of the VIS are good. A publication about the psychometrics is underway.

#### O-AS: to assess agoraphobic avoidance and distress

The Oxford Agoraphobic Avoidance Scale (O-AS)^49^ is an 8-item questionnaire assessing avoidance and associated distress. The scale has demonstrated good internal reliability, test–retest reliability, and validity.

#### OPDBQ: to assess safety-seeking behaviours and in situ-defences

The Oxford Paranoia Defence Behaviours Questionnaire (OPDBQ) is a newly developed questionnaire (Lambe et al., *in prep*) assessing avoidance in the past two weeks (Part 1; 12 items) and in situ-defences (Part 2; 20 items) which are strategies people use to keep themselves safe.

#### EofE: to assess access to alternative explanations

The Explanations of Experience (EofE) Interview [50] is a brief interview in which participants are asked to detail the key evidence they have in support of their main (distressing) belief. They are then asked: ‘Can you think of any other explanations for what you have described? Are there any other reasons, besides what you have said, that could possibly account for these experiences/ this evidence even if you think they are very unlikely?’ Alternative explanations are scored ‘Yes’ or ‘No’ indicating the presence/absence of flexibility.

#### FaST: to assess fast and slow thinking biases

The Fast and Slow Thinking (FaST) Questionnaire [51] is a 10-item self-report questionnaire assessing fast and slow thinking biases related to paranoid thoughts. The questionnaire involves 5-item assessing fast thinking and 5-item assessing slow thinking.

#### MADS-PM: to assess belief flexibility

The Maudsley Assessment of Delusions Schedule – Probability of Being Mistaken (MADS-PM) is a commonly used ‘Yes’ or ‘No’ item assessing whether people consider the possibility that they are being mistaken about their persecutory delusion [52].

#### QPR: to assess personal recovery

The 15-item questionnaire about the process of recovery (QPR) assesses personal recovery [53]. The QPR showed internal consistency, construct validity and reliability.

#### BRS: to assess resilience

The Brief Resilience Scale (BRS) is a 6-item questionnaire of resilience that was found to have good psychometric properties [54].

#### Qualitative interviews

Semi-structured qualitative interviews will be conducted with participants regarding their experiences with both therapies. The interviews that are conducted post-therapy will mainly focus on patients’ experiences with (1) collaborating with their therapist and peer counsellor (if Feeling Safe-NL) and effect on their recovery and (2) self-monitoring and visual feedback using the m-Path application. Interviews conducted 1-year after therapy completion will mainly focus on participants’ experiences with long-term recovery. Therapists and peer counsellors will also be interviewed after seeing several participants. The interviews will mainly focus on (1) their collaboration in the Feeling Safe-NL Programme and (2) using the m-Path application. Participants will be interviewed until saturation is achieved on all topics.

### Plans to promote participant retention and complete follow-up {18b}

This project team is committed to a recovery-oriented approach, and everyone working on the trial will be trained to ensure respectful and meaningful interactions with patients and community mental health staff. Trial assessors can visit participants at their local community mental health team or at their homes. Participants are also compensated for their time and travel. We also know from previous trials that patients remain motivated and generally like to contribute to this type of study [65]. Additionally, the Feeling Safe trial had high follow-up rates with less than 10% drop-out [5].

### Data management {19}

Questionnaire data (collected at 0-, 6-, 12-, and 18-month follow-up) will be collected in the Data Manager module of Research Manager in agreement with local mental healthcare institutions and VU University Amsterdam guidelines. Data from self-monitoring measures will be collected using the GDPR compliant platform m-Path of the KU Leuven (www.m-path.io). Audio recordings of the therapy sessions (for supervision and fidelity ratings) and qualitative interviews will also be stored in agreement with the VU University Amsterdam guidelines using the program Research Drive.

### Confidentiality {27}

Participants are given a research number (before randomization) and a randomization number (after randomization). No participant information is stored in the Research Manager. Participant information is kept on a separate and secured drive in a recruitment file with a password. This data is only accessible by the principal investigator, the project coordinator and trial assessors. In line with WMO guidelines, data will be stored for 15 years after the last intervention has been provided. We will save the key file about the participants for the entire period of 15 years for safety reasons.

### Plans for collection, laboratory evaluation and storage of biological specimens for genetic or molecular analysis in this trial/future use {33}

N/a. The trial involves an investigation of two psychological interventions using questionnaires and interviews.

## Statistical methods

### Statistical methods for primary and secondary outcomes {20a}

The primary continuous outcomes for mental wellbeing will be analyzed with linear mixed models (LMM) with outcome measurement (at the three follow-up timepoints) as the dependent variable and based on the intention-to-treat (ITT) principle. Baseline values will be added as covariates, time as a categorical variable, and treatment condition as a fixed effect. The regression coefficient of the LMM will be used as the between-group effect size (Feeling Safe-NL vs formulation-based CBTp) since this is the estimated difference in results between the groups.

The secondary continuous outcomes for the persecutory delusion, general paranoid ideation, patient-chosen therapy outcomes and activity will also be analyzed with LMM with outcome measurements (at the three follow-up timepoints) as the dependent variable and based on the intention-to-treat (ITT) principle. Baseline values will be added as covariates, time as a categorical variable, and treatment condition as a fixed effect. Between-group effect sizes (Feeling Safe-NL vs formulation-based CBTp) will be derived from the regression coefficient of the LMM. Outcomes for conviction of the main persecutory delusion (PSYRATS-DS) will also be dichotomized with recovery being defined as conviction falling below 50%. Logistic generalized estimating equations (GEE) with exchangeable correlation structure ITT analysis will be used to examine the effects on dichotomous outcomes, since GEE is reported to be a better estimator of effects in dichotomous outcomes than LMM [66]. Effects for Feeling Safe-NL vs formulation-based CBTp will be computed for “condition” (overall effect) and with interaction effects between time and condition for post-therapy (6-month follow-up; change during the treatment period) and for 12- and 18-month follow-up (change during the follow-up periods).

#### Mediation

We will also carry out parallel mediation analysis using the PROCESS macro for SPSS [67] to assess the effect of changes in maintenance factors of threat beliefs (e.g. worry, low self-esteem, anomalous experiences, insomnia, safety-seeking behaviours or defences, and trauma imagery), personal recovery and resilience on both mental wellbeing and severity of the persecutory delusion using the assessments at baseline, and 6 (post-therapy), 12- and 18-month follow-up. We will also assess whether the therapeutic relationship mediates changes from baseline to 6 months (post-therapy) and vise-versa.

Both (1) cost-effectiveness analysis (CEA) and (2) cost-utility analysis (CUA) will be conducted alongside the randomized trial. The outcome is Treatment Response (TR) defined as a clinically relevant change, in the CEA. In the CUA the outcome will be QALYs as estimated by the EQ-5D-5L using Dutch tariffs. Since we need to assign costs to meaningful outcomes, we use both QALYs and clinically relevant change defined using the primary outcome of mental wellbeing. For a clinically relevant change, we use Jacobson & Truax reliable change. For mental wellbeing measured with the WEMWBS, this corresponds to an improvement of 5 points. We calculated this as follows: SE means = 8.2 * square root (10.83) = 3.38; reliable change index: (2 × (3.38)^2^) = 5) of 5 points in WEMWBS mental wellbeing, based on studies on the WEMWBS and the Feeling Safe Trial [5, 28, 68]. Four types of costs will be considered: (1) intervention costs, (2) healthcare costs, (3) patients’ costs for making round trips to health services and for informal care, (4) costs stemming from productivity losses due to absenteeism and lesser efficiency while at work, both in paid and unpaid work. Costs, in Euros, will be estimated by multiplying units of health service (e.g. outpatient contacts, therapy sessions, hospital days) with their standard unit cost price as reported in the Dutch guideline for health economic evaluation indexed to the year 2022. The trial’s baseline balance between the Feeling Safe-NL and the formulation-based CBTp conditions will be assessed for both costs and outcomes and disbalances, if any, will be handled using appropriate regressions-based adjustments [69, 70]. Since the health economic follow-up at 18 months post-baseline does exceed 1 year, costs and effects will be discounted. Cumulative costs and health gains will be computed with the area under the curve method (or linear interpolation) to obtain cumulative estimates for costs, TR and QALY gains as accrued over the 18-month follow-up. The incremental cost-effectiveness ratio (ICER) will be computed to obtain the incremental costs per treatment responder and per QALY gained. To simultaneously evaluate both costs and outcomes, seemingly unrelated regression equations (SURE) models will be used. Because costs are usually non-normally distributed the SURE models will be bootstrapped (2500 times). Missing data will be imputed using predictive mean matching nested in nonparametric bootstraps of SURE models of costs and effects for intention-to-treat (ITT) analysis, as recommended by Brand and colleagues [71]. To visually present stochastic uncertainty, the bootstrapped ICERs will be plotted on the ICER plane. For decision-making purposes, the ICER acceptability curve will be plotted for various willingness-to-pay (WTP) ceilings for making judgments whether the Feel Safe-NL intervention offers good value for money relative to the formulation-based CBTp comparator condition. One-way sensitivity analyses directed at uncertainty in the main cost drivers (e.g. costs of hospital re-admissions) and outcomes (e.g. under different imputations) will be performed to assess the robustness of our findings. Both the analysis and reporting of the research findings will conform to the (extended) CONSORT and CHEERS-2022 statements [72].

##### Qualitative parameters

Inductive Thematic Analysis [73, 74] will be used to analyze themes in the central themes including the target interventions, collaboration, self-monitoring and the process of recovery. These will be coded using labels for participants’, therapists’, and peer counsellors’ words. When interviews are analyzed for the primary themes, the codes of the interviews will be analyzed together to identify the overall emerging sub-themes within the main themes.

### Interim analyses {21b}

N/a. Analyses are conducted at the end of the trial.

### Methods for additional analyses (e.g. subgroup analyses) {20b}

N/a. There is no plan for any additional analyses.

### Methods in analysis to handle protocol non-adherence and any statistical methods to handle missing data {20c}

Missing outcome data in the clinical effectiveness analyses will be handled by the LMM analysis (i.e. given its robustness to missing data) [75]. In the economic evaluations (i.e. CEA and CUA), missing cost and outcome (e.g. QALYs) data will be handled using single imputations nested in 5000 non-parametric bootstraps.

### Plans to give access to the full protocol, participant-level data and statistical code {31c}

Data will be made accessible after an embargo period (completion of PhD project), for verification purposes, safety monitoring, and future research with some restrictions: (1) the approval of the participants as indicated in the consent form, (2) conditions related to data security, (3) quality assessment of the proposed research, (4) collaboration in using the data set. Potential restrictions for data access are evaluated with the privacy experts at the various partner organizations to ensure the protection of the study population, and even if the data are not made available following request, we will make the metadata describing our research publicly available through registration in PURE and any other outputs that do not involve data collected about the participants.

## Oversight and monitoring

### Composition of the coordinating centre and trial steering committee {5d}

The trial is run by the principal investigator, project coordinator (PhD) and trial assessors who meet on a (bi-) weekly basis. There is also a meeting with the trial steering committee every three months including members of the Oxford Cognitive Approaches to Psychosis (O-CAP) research group, trial supervisors, peer counsellors, local principal investigators of the participating mental healthcare institutions, and the randomization bureau.

### Composition of the data monitoring committee, its role and reporting structure {21a}

N/a. The interventions are proven to be acceptable and are used in clinical practice.

### Adverse event reporting and harms {22}

The interventions (formulation-based CBTp, Feeling Safe, and peer counselling) are found to be acceptable and are used in clinical practice. Therefore, no major adverse events related to the interventions are expected. Nor is there any expectation that combining Feeling Safe and peer counselling would result in adverse outcomes. Adverse events reported spontaneously by the participant or observed by the investigators or staff will be recorded. If a serious adverse event occurs, the responsible therapist (and peer counsellor) takes action. The principal investigator will report the serious adverse event through the web portal to the accredited medical ethics committee that approved the protocol within 7 days of first knowledge for serious adverse events that result in death or are life-threatening followed by a period of a maximum of 8 days to complete the initial preliminary report. All other serious adverse events will be reported within a period of a maximum of 15 days after the investigator has first knowledge of the serious adverse events. An elective hospital admission, suicidal thoughts, self-harm, and crisis contacts with mental healthcare professionals will not be considered as a serious adverse event as this regularly occurs within this population. Somatic illnesses will also not be considered as a serious adverse event as this is unrelated to participation in the study.

### Frequency and plans for auditing trial conduct {23}

N/a. A trial steering group was established instead of a data monitoring committee.

### Plans for communicating important protocol amendments to relevant parties (e.g. trial participants, ethical committees) {25}

Important protocol modifications (e.g. changes to eligibility criteria, outcomes, analyses) will be submitted to the relevant medical ethics committee for review and communicated during the meeting with the trial steering committee and during assessments with participants, if applicable.

### Dissemination plans {31a}

Results will be communicated through publications in journals and presentations at established conferences. Participants who have agreed to receiving the results will be sent a newsletter. Broader dissemination of the findings will occur through the organization Cognition & Psychosis (https://www.gedachtenuitpluizen.nl). This organization is dedicated to disseminating psychological therapies for people with threat beliefs and/or hallucinations. It is also the central training institute in The Netherlands for psychological therapies in psychosis. Materials will be made available to a wide group of people using the organization’s website and newsletter. With the dissemination funding from the sponsor, we can also develop a training course and associated materials.

## Discussion

Previous research on the translational and modular Feeling Safe Programme found larger treatment effects than are usually observed for formulation-based CBTp. The current trial aims to enhance this work by (1) combining Feeling Safe with peer counselling; (2) by introducing trauma imagery as a treatable maintenance factor; and (3) introducing visual feedback of self-monitoring to empower and support people in shared decision-making and tracking of progress. Feeling Safe-NL is the first program for patients with persecutory delusions in which therapists and professional peer counsellors collaborate to improve mental wellbeing and feelings of safety. Therefore, the program moves towards a dual approach to therapy involving systematically removing the factors that hamper recovery while synergistically promoting personal recovery. The Feeling Safe-NL Programme addresses the key problems people with persecutory delusions are seeking help for regardless of diagnostic category. The current trial aims to determine the effectiveness and cost-effectiveness of the Feeling Safe-NL Programme above that of the guideline intervention formulation-based CBTp. The potential is a therapy that may be more effective and cost-effective on the long-term. Outcomes are expected in 2026.

## Trial status

Protocol version 2 (21–12-2022). Recruitment started on the 14th of March 2022 and is expected to be completed in March 2025.

## Data Availability

The Principal Investigator, project coordinator (PhD) and trial assessors will have access to the final trial dataset. Data required to support the protocol can be supplied on request with some restrictions (see SPIRIT item 31c).

## Abbreviations

BCSS: Brief Core Schema Scales
BRS: Brief Resilience Scale
CBTp: Cognitive Behaviour Therapy for psychosis
CEA: Cost-Effectiveness Analysis
CHOICE: CHoice of Outcome In Cognitive behaviour therapy for psychosEs
CRF-S: Counsellor Rating Form-Short
CUA: Cost-Utility Analysis
DSM: Diagnostic and Statistical Manual of Mental Disorders
DWQ: Dunn Worry Questionnaire
EQ-5D-5L: EuroQol 5-Dimension 5-Level
EofE: Explanations of Experience
FaST: Fast and Slow Thinking
GDPR: General Data Protection Regulation
GEE: Generalized Estimating Equations
HRQoL: Health-Related Quality of Life
ICER: Incremental Cost-Effectiveness Ratio
ISI: Insomnia Severity Index
ITT: Intention-To-Treat
LMM: Linear Mixed Models
MADS-PM: Maudsley Assessment of Delusions Schedule - Possibility of being Mistaken
O-AS: Oxford Agoraphobic Avoidance Scale
O-CAP: Oxford Cognitive Approaches to Psychosis
OPDBQ: Oxford Paranoia Defence Behaviours Questionnaire
PCL-5: Posttraumatic Stress Disorder Checklist for DSM-5
PSYRATS-DS: Psychotic Symptom Rating Scales-Delusions Subscale
QALY: Quality-Adjusted Life Years
QPR: Questionnaire about the Process of Recovery
RCT: randomized controlled trial
R-GPTS: Revised-Green et al. Paranoid Thoughts Scale
SPIRIT: Standard Protocol Items Recommendations for Interventional Trials
SURE: Seemingly Unrelated Regression Equations
TALE: Trauma And Life Events checklist
TIC-P: Treatment Inventory of Costs in Patients with psychiatric disorders
TR: Treatment Response
TSQ: Trauma Screening Questionnaire
VIS: Voices Impact Scale
WAI-SR: Working Alliance Inventory-Short Form Revised
WEMWBS: Warwick-Edinburgh Mental Well-Being Scale
WTP: Willingness-To-Pay
