# Correction: Testing the combination of Feeling Safe and peer counselling against formulation-based cognitive behaviour therapy to promote psychological wellbeing in people with persecutory delusions: study protocol for a randomized controlled trial (the Feeling Safe-NL Trial)

**DOI:** 10.1186/s13063-023-07750-x

**Published:** 2023-12-18

**Authors:** Eva Tolmeijer, Felicity Waite, Louise Isham, Laura Bringmann, Robin Timmers, Arjan van den Berg, Hanneke Schuurmans, Anton B. P. Staring, Paul de Bont, Rob van Grunsven, Gert Stulp, Ben Wijnen, Mark van der Gaag, Daniel Freeman, David van den Berg

**Affiliations:** 1 Department of Clinical Psychology, VU University and Amsterdam Public Health Research, Amsterdam, The Netherlands; 2 Department of Psychosis, Parnassia Psychiatric Institute, The Hague, The Netherlands; 3 Department of Experimental Psychology, University of Oxford and Oxford Health NHS Foundation Trust, Oxford, UK; 4 Department of Psychometrics and Statistics, University of Groningen, Groningen, The Netherlands; 5 Voice-Hearing Support and Recovery-Team, RIBW Nijmegen and Rivierenland, Nijmegen, The Netherlands; 6 University of Applied Sciences Nijmegen, Nijmegen, The Netherlands; 7 Department of Health, Wellbeing and Sport, Zadkine College Rotterdam, Rotterdam, The Netherlands; 8 Mental Health Organizations Oost Brabant, Boekel, The Netherlands; 9 ABC Department for First Episode Psychosis, Altrecht Psychiatric Institute, Utrecht, The Netherlands; 10 Department of Sociology, University of Groningen, Groningen, The Netherlands; 11 Centre of Economic Evaluation and Machine Learning, Trimbos Institute (Netherlands Institute of Mental Health and Addiction), Utrecht, The Netherlands

**Correction: Trials 24, 644 (2023)**

**https://doi.org/10.1186/s13063-023-07661-x**

Following publication of the original article [1], we have been notified that the name of the 8^th^ author had been incorrectly written.

Originally published name: Anton B. P. (Tonnie) Staring

Correct name: Anton B. P. Staring

The original article has been corrected.
